# Next Steps for Interventions Targeting Adolescent Dietary Behaviour

**DOI:** 10.3390/nu12010190

**Published:** 2020-01-09

**Authors:** Charlotte E. L. Evans

**Affiliations:** School of Food Science and Nutrition, University of Leeds, Leeds LS2 9JT, UK; c.e.l.evans@leeds.ac.uk

**Keywords:** intervention, adolescent, dietary behaviour, education, environment, nutrition policy, sugary drinks, vegetables

## Abstract

Adolescents in many countries consume poor quality diets that include high intakes of sugary drinks and fast food and low intakes of vegetables. The aims of this Special Issue on adolescent dietary behaviour were to identify methods and approaches for successful interventions to improve diet quality in this age group and identify at risk subgroups that need particular attention. In total, 11 manuscripts were published in this Special Issue—three qualitative studies which included a systematic review, five cross-sectional studies and three quantitative evaluations of interventions. This Editorial discusses the contribution of the studies and provides suggestions to improve the success of future interventions in adolescents. It is important that adolescents are involved in the design of interventions to improve social and cultural acceptability and relevance. Interventions targeting schools or communities framed within a larger food system such as issues around climate change and the carbon footprint of food may improve engagement. Furthermore, targeting adolescents in areas of lower deprivation is a priority where diet quality is particularly poor. Potentially successful interventions also include environmental policies that impact on the cost and marketing of food and drinks, although evaluations of these were not included in this issue.

## Next Steps for Interventions Targeting Adolescent Dietary Behaviour

Diets of adolescents in many countries are of poor quality when compared to those of younger and older age groups; higher in fats and sugars and lower in vegetables [1]. Intakes of fast food outside the home are often higher [2]; members of this age group are more likely to start engaging in risky behaviour including drinking alcohol and they are usually concerned with the views and opinions of their peers as well as their family [3]. This is an important age group to target as poor diet habits may persist into adulthood and increase the risk in the future of non-communicable diseases such as type 2 diabetes, cardiovascular disease and cancers [4]. These adolescents are also future parents and, therefore, good dietary habits can potentially be passed on to the next generation. This Special Issue focuses on evidence to help target and improve adolescent diet either through individual intervention or through environment-based policies. The issue includes three types of manuscripts which provide a wealth of information to inform future intervention programmes and policies targeting this age group. These are discussed below in turn, followed by a synthesis of the learnings from the body of evidence presented. First, the qualitative research from three papers based on alcohol (a systematic review), wholegrain foods (individual interviews) and school food (stakeholder focus groups) is reviewed. Second, five cross-sectional studies that include information on diet quality and dietary patterns in adolescents from five different countries are discussed. Third, three interventions based in the US and Canada targeting adolescents are examined.

Qualitative research is an important, although under-utilised, part of the process of designing successful interventions and can ensure that evaluation trials are more effective and efficient [5]. The review of qualitative research on adolescent behaviour and opinions related to alcohol in this issue was enlightening. It highlighted the difficulties and complexities inherent in influencing adolescent behaviour which includes dietary behaviour [3]. The tensions between restraint and fun, which may be due to acting on family values and beliefs versus those held by groups of peers, are high in this age group. Conflicting behaviour and views are common, leaving adolescents in challenging positions where they are expected to join in and drink and eat fast food but still maintain a healthy lifestyle and not get drunk or be overweight [3]. The review also highlighted the high rate of personal problems at this age, which may well mean that immediate mental health issues are far more of a priority than long-term physical health. Further and much-needed qualitative work from secondary schools highlighted the fact that most adolescents would choose unhealthier foods if available but primarily wanted food that was easy to purchase and carry around [6]. The challenge is to design one-pot portable meals that, unlike fast food, are not deep fried and do contain useful amounts of vegetables. Innovative methodology using a SenseCam camera to collect data reported on barriers and facilitators to improve wholegrain intake revealed that knowledge is often patchy but poor availability of healthy products aimed at this age group made it particularly difficult to make quick healthy choices [7]. Taste was mentioned as an important factor in all the qualitative studies when referring to food but not for alcohol, highlighting the importance of peers and the differences between drinking and dietary behaviours.

Cross-sectional research is useful for identifying specific foods and nutrients that are far from optimal, particularly poor or good dietary patterns and disparities in diet between specific subgroups of the adolescent population. We included a range of populations in this Special Issue, namely adolescents from Belgium [8], China [9], Norway [10], Poland [11] and the UK [12]. Different types of foods rather than nutrients were more likely to be reported, and the top dietary behaviours reported by all of the five cross-sectional studies were sugary drinks or foods and vegetables. Wholegrain foods, fruit, fast food or school food and skipping breakfast were also mentioned by at least two studies. A number of factors had an impact on sugary foods and drinks; consumption of sugary drinks was reported to rise with age [11], was more common in adolescent boys [10] and more common in less educated or lower social class households [8,10]. For example, in Belgium, authors also reported that amounts of sugary drinks were around 50% higher and wholegrain foods were 50% lower in households where education was lower [8]. Some of the studies also reported that unhealthy behaviours clustered together, pointing to the fact that interventions targeting more than one behaviour might be more effective [9,10,11]. Dietary data from the UK indicated that high intakes of extrinsic or added sugars are driven mainly by high intakes of sugary drinks in this age group as well as cakes, biscuits and confectionery. Furthermore, higher intakes of pasta and rice, wholemeal bread and fish were associated with lower sugar consumption. The very low proportion of adolescents meeting the recommendation (4%) and the findings that the most nutritious diets in the UK are for categories of 10–15% extrinsic sugars indicate that dietary patterns need to change dramatically in many countries in order to meet current guidelines. Although sugary drinks are a key driver of high free sugar intake, it is unlikely that solely targeting this food group will be sufficient to improve overall diet quality.

Three papers in this Special Issue reported on interventions aimed at adolescents to improve diet. One looked at how a small number of education classes on sugar literacy increased adolescents’ intention to limit sugar intake and their confidence to read food labels [13]. A second study tested a nudge-based intervention to increase vegetables that had a small impact on vegetable intake [14]. A third study was a two-part intervention based on education followed by an adolescent-designed promotional activity focussed on food choices and food waste [15]. This study was successful in reducing vegetable waste in adolescents. The intervention studies published here support the need for interventions to provide high quality information, the skills to interpret complex dietary information and the framing of nutritional problems in a wider food system. Improving dietary behaviour in this age group, such as reducing portion sizes of energy dense food and encouraging higher intakes of plant-based diets, may be more likely to engage adolescents if linked to larger issues such as climate change. This could include interventions to reduce portion size to reduce food waste and increase high-fibre nutritious plant foods to reduce food’s carbon footprint. Co-designed and adolescent-led interventions are likely to be more attuned to adolescent needs and values and therefore are potentially more effective.

It is worth mentioning what is missing from this Special Issue. No manuscripts were submitted evaluating interventions targeting the wider environment, including marketing and offering incentives or disincentives such as a subsidy (on vegetables) or a tax or levy (on sugary drinks or foods). In the UK, Public Health England have reported that as a result of the recent levy, half of sugary drinks have been reformulated to contain lower levels of sugars and this is likely to have a disproportionate impact on behaviour in adolescents as they are the largest consumers of sugary drinks. Furthermore, a reduction in marketing of foods high in fats, sugars and salt is identified by the WHO as a priority area, which is also likely to impact this age group more than others [16] and has already been implemented in some countries in Europe but was not featured here.

In conclusion, this Special Issue provides insights into designs of interventions and policies that have the potential to improve adolescent dietary behaviour. Policies that frame the issues within a larger food system and that target multiple eating behaviours may be more likely to be successful at changing overall diet quality and, consequently, health in this age group. Education is still needed despite nutrition education being part of the curriculum in schools. Furthermore, it is important that interventions take into account aspects of young people’s emotional, social and cultural lives if they are to be successful. Interventions targeting adolescents in more deprived regions are particularly needed due to the inequalities in diet. Digital and online platforms are important sources of information in this age group, sources that have been implemented to improve dietary behaviour albeit with limited success so far [17]. This approach could have potential but is fraught with ethical issues due to problems with fake news and misinformation that particularly target this age group. It is essential that adolescents are at the heart of co-designing these complex interventions as it will be difficult for others to truly understand the social and cultural issues involved.

## References

[1] Llaurado E., Albar S.A., Giralt M., Sola R., Evans C.E. (2015). The effect of snacking and eating frequency on dietary quality in british adolescents. Eur. J. Nutr..

[2] Taher A.K., Evans N., Evans C.E. (2019). The cross-sectional relationships between consumption of takeaway food, eating meals outside the home and diet quality in british adolescents. Public Health Nutr..

[3] Scott S., Elamin W., Giles E.L., Hillier-Brown F., Byrnes K., Connor N., Newbury-Birch D., Ells L. (2019). Socio-ecological influences on adolescent (aged 10–17) alcohol use and unhealthy eating behaviours: A systematic review and synthesis of qualitative studies. Nutrients.

[4] Craigie A.M., Lake A.A., Kelly S.A., Adamson A.J., Mathers J.C. (2011). Tracking of obesity-related behaviours from childhood to adulthood: A systematic review. Maturitas.

[5] O’Cathain A., Thomas K.J., Drabble S.J., Rudolph A., Hewison J. (2013). What can qualitative research do for randomised controlled trials? A systematic mapping review. BMJ Open.

[6] McSweeney L., Bradley J., Adamson A.J., Spence S. (2019). The ‘voice’ of key stakeholders in a school food and drink intervention in two secondary schools in ne england: Findings from a feasibility study. Nutrients.

[7] Kamar M., Evans C., Hugh-Jones S. (2019). Factors influencing british adolescents’ intake of whole grains: A pilot feasibility study using sensecam assisted interviews. Nutrients.

[8] Desbouys L., De Ridder K., Rouche M., Castetbon K. (2019). Food consumption in adolescents and young adults: Age-specific socio-economic and cultural disparities (belgian food consumption survey 2014). Nutrients.

[9] Sun S., He J., Fan X. (2019). Mapping and predicting patterns of chinese adolescents’ food preferences. Nutrients.

[10] Skeie G., Sandvaer V., Grimnes G. (2019). Intake of sugar-sweetened beverages in adolescents from troms, norway-the tromso study: Fit futures. Nutrients.

[11] Myszkowska-Ryciak J., Harton A., Lange E., Laskowski W., Gajewska D. (2019). Nutritional behaviors of polish adolescents: Results of the wise nutrition-healthy generation project. Nutrients.

[12] Lai H.T., Hutchinson J., Evans C.E.L. (2019). Non-milk extrinsic sugars intake and food and nutrient consumption patterns among adolescents in the uk national diet and nutrition survey, years 2008–16. Nutrients.

[13] Santalo M.I., Gibbons S., Naylor P.J. (2019). Using food models to enhance sugar literacy among older adolescents: Evaluation of a brief experiential nutrition education intervention. Nutrients.

[14] Mistura M., Fetterly N., Rhodes R.E., Tomlin D., Naylor P.J. (2019). Examining the efficacy of a ‘feasible’ nudge intervention to increase the purchase of vegetables by first year university students (17–19 years of age) in british columbia: A pilot study. Nutrients.

[15] Prescott M.P., Burg X., Metcalfe J.J., Lipka A.E., Herritt C., Cunningham-Sabo L. (2019). Healthy planet, healthy youth: A food systems education and promotion intervention to improve adolescent diet quality and reduce food waste. Nutrients.

[16] World Health Organization (2019). Reducing the Impact of Marketing of Foods and Non-Alcoholic Beverages on Children.

[17] Sharps M.A., Hetherington M.M., Blundell-Birtill P., Rolls B.J., Evans C.E. (2019). The effectiveness of a social media intervention for reducing portion sizes in young adults and adolescents. Digit Health.

